# Risk of Misdiagnosis in Spinal Hypertrophic Pachymeningitis: A Report of Two Cases

**DOI:** 10.7759/cureus.60546

**Published:** 2024-05-18

**Authors:** Hiroshi Kageyama

**Affiliations:** 1 Neurological Surgery, Shin-Kuki General Hospital, Kuki, JPN

**Keywords:** spinal tumor, meningioma, igg4-related disease, herniated disc, hypertrophic pachymeningitis

## Abstract

Hypertrophic pachymeningitis (HP) is a rare inflammatory disease of the central nervous system. It typically manifests in the cranium; cases involving the spinal cord are rare (8.6%). This report includes two cases of spinal HP encountered among 666 spinal operative cases. The purpose of this study is to present the initial imaging findings, final diagnosis, and course of treatment in these two cases of spinal HP and to present the possible risk of misdiagnosis with a literature review. In case 1, a 69-year-old female presented with back pain. The initial radiological diagnosis with magnetic resonance imaging (MRI) was a meningioma. However, her blood test showed a mild elevation of C-reactive protein level (3.16 mg/dL), with positive IgG4 and myeloperoxidase anti-neutrophil cytoplasmic antibody results, suggesting an autoimmune disease. We performed a biopsy of the thickened dura and an expansive duraplasty. Serological and pathological diagnosis suggested IgG4-related HP. In case 2, a 67-year-old male presented with bilateral thigh pain. MRI revealed a mass resembling a disc hernia at the L2/3 intervertebral level. The mass was surgically removed. Pathological examination and cerebrospinal fluid analysis confirmed the diagnosis of HP associated with IgG4-related disease. In both cases, immunosuppressive therapy was administered, and follow-up MRI scans revealed the disappearance of the mass. The study concludes that a spinal HP can potentially be misdiagnosed when its images resemble those of tumors or disc hernias owing to its rarity.

## Introduction

Hypertrophic pachymeningitis (HP) is a relatively rare inflammatory disease of the central nervous system, with an incidence rate of 9.49 per one million in Japan [1]. Not any large-scale epidemiological studies have been conducted yet in global [2]. HP was defined as a condition with thickening of the cranial or spinal dura mater with inflammation [1]. It typically manifests in the cranium, and spinal cases are exceptionally rare (8.6%) and may present with mass effect [1,3,4].

HP is broadly classified into idiopathic and secondary cases. Secondary cases include infection (syphilis, tuberculosis, fungi, etc.), autoimmunity-mediated disease (anti-neutrophil cytoplasmic antibody (ANCA)-associated vasculitis, sarcoidosis, rheumatoid arthritis, IgG4-related disease (IgG4-RD)), and others [1,4,5]. Until the first half of the 20th century, infectious HP was common, but autoimmunity-mediated HP has been recently reported in many cases [1,3,4]. A nationwide survey of 159 cases of HP in Japan over a five-year period beginning in 2005 showed that idiopathic, ANCA-associated vasculitis and IgG4-RD accounted for most cases (86.8 %), with only four cases of infection [1].

We present cases in which the initial imaging diagnosis was neoplastic lesions or degenerative disease, and the final diagnosis was spinal HP, along with a literature review.

## Case presentation

We conducted a retrospective chart review of operative cases related to spinal diseases over the past five years (from July 2018 to June 2023). From this pool, we identified patients who had been diagnosed with HP. We reviewed 666 operative spinal cases in Shin-Kuki General Hospital, among which we encountered two cases of spinal HP.

Case 1

**Figure 1:**
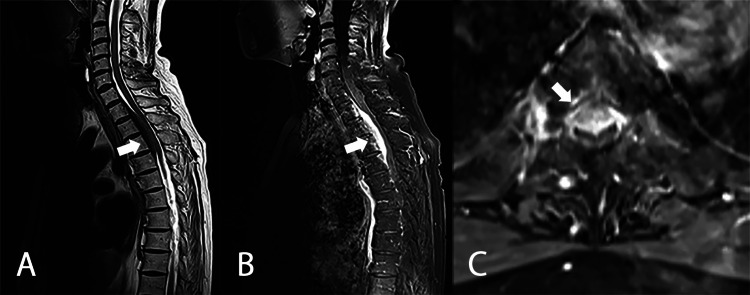
Initial thoracic MRI of case 1 A: T2WI sagittal; B: T1WI contrast enhancement sagittal; C: T1WI contrast enhancement axial. Extramedullary mass lesion located ventral to the spinal cord in T1-T4 vertebral level. The initial radiological diagnosis was a meningioma MRI: magnetic resonance imaging

A 69-year-old female with a medical history of autoimmune hepatitis and left breast cancer presented with severe back pain without paresis or sensory disturbances in her lower extremities for four months. Magnetic resonance imaging (MRI) of the thoracic spine revealed an intradural extramedullary mass with strong contrast enhancement on the ventral side of the spinal cord at the T1-T4 vertebral level (Figure 1). The initial radiological diagnosis was a thoracic meningioma ventral to the spinal cord, which led to a consultation with our team.

However, her condition was accompanied by a mild elevation in the C-reactive protein (CRP) level (3.16 mg/dL), prompting the consideration of HP. Further blood tests revealed markers suggesting an autoimmune disease (IgG4, 139 mg/dL; myeloperoxidase anti-neutrophil cytoplasmic antibody (MPO-ANCA)-positive) (Table 1).

**Table 1:** Laboratory findings of case 1 IST: immunosuppressive therapy; AST: aspartate aminotransferase; ALT: alanine aminotransferase; BUN: blood urea nitrogen; MPO-ANCA: myeloperoxidase anti-neutrophil cytoplasmic antibodies; PR3-ANCA: proteinase-3-anti-neutrophil cytoplasmic antibodies; SS: Sjogren syndrome; RNP: ribonucleoprotein; ACE: angiotensin-converting enzyme; sIL2R: soluble IL2 receptor; WBC: white blood cell; RBC: red blood cell

Investigations	Initial checkup	After IST	Normal range
Biochemistry/immunology			
Total protein (g/dL)	8.2	5.8	6.6-8.1
Serum albumin (g/dL)	3.8	3.3	4.1-5.1
AST (U/L)	31	22	7-38
ALT (U/L)	15	22	4-43
BUN (mg/dL)	16.3	21.9	8.0-20.0
Serum creatinine (mg/dL)	0.71	0.69	0.60-1.20
C-reactive protein (mg/dL)	3.16	0.05	0-0.3
IgG (mg/dL)	1949	865	820-1,400
IgA (mg/dL)	417	141	90-400
IgM (mg/dL)	76	20	52-270
IgG4 (mg/dL)	139	44	11-121
C3 (mg/dL)	170		80-140
C4 (mg/dL)	28.7		11-34
Antinuclear antibody (titer)	1:40		<40
MPO-ANCA (U/mL)	9.9		<3.5
PR3-ANCA (U/mL)	<0.6		<3.5
Anti-SS-A antibody	Negative		
Anti-SS-B antibody	Negative		
Anti-Sm antibody	66.6		<6.9
Anti-U1 RNP antibody	Negative		
T-SPOT.TB	Negative		
ACE (U/L)	14.0		7.0-25.0
sIL2R antibody (U/ml)	189		122-496
Complete blood cell count			
WBC (/µL)	7,300	5,500	3,600-8,000
Neutrophil (%)	72	73	37.0-72.0
Lymphocyte (%)	20	19	19.0-49.0
Monocyte (%)	7	6	2.0-11.0
Eosinophil (%)	1	2	0-5.0
RBC (×10^4^/µL)	386	363	380-480
Hemoglobin (g/dL)	10.9	10.1	12.0-16.0
Hematocrit (%)	33.7	32.6	36.0-48.0
Platelet (×10^4^/µL)	35.3	19.8	12.0-40.0

We consulted a neurologist to determine whether HP diagnostic treatment (e.g., immunosuppression) was possible. Based on the blood sample results, the neurologists believed that there was a 90% probability of autoimmune-mediated HP, but there could be other conditions, such as malignant lymphoma, that temporarily resolved with steroid administration. For the treatment, a pathological diagnosis of the mass lesion in the spinal canal was required. We performed a thoracic laminectomy (T1-T4), biopsied the thickened dura on the dorsal side, and performed expansive duraplasty.

**Figure 2:**
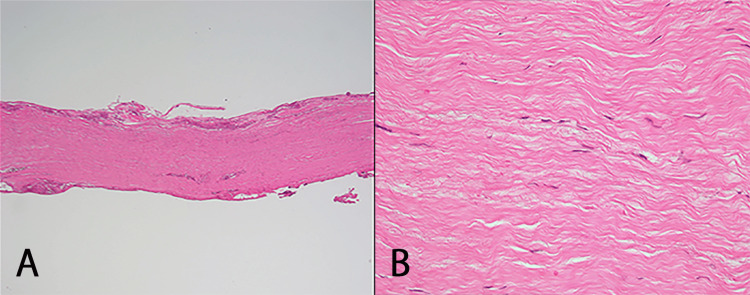
Hypertrophic dura specimen with hematoxylin-eosin stain A: weak magnification (40×); B: strong magnification (400×). Collagenous fiber tissue with laminar thickening. Consistent with pachymeningitis, including post-inflammatory scar tissue with little inflammatory cellular infiltration or vascularity

**Figure 3:**
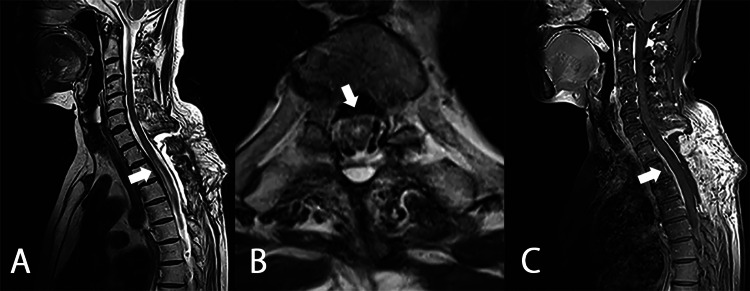
Thoracic MRI of case 1, four weeks after operation and immunosuppressive therapy A: T2WI sagittal; B: T1WI with contrast enhancement axial section; C: T1WI with contrast enhancement sagittal section. The mass has disappeared (white arrows) MRI: magnetic resonance imaging

The pathological findings were consistent with HP but not lymphoid tumors (Figure 2). With a diagnosis of immune-mediated HP (possible IgG4-related HP) and spinal cord compression by the residual mass on the ventral side of the spinal cord, the neurologist of our hospital decided to start treatment which included immunosuppressive therapy with rituximab (600 mg/week) and prednisolone (started at 50 mg/day and tapered to 15 mg/day over four weeks), resulting in the disappearance of the mass four weeks later (Figure 3). Her laboratory test improved (WBC 5500 /μL, CRP 0.05 mg/dL). The patient was discharged without pain medications and was able to walk on her own. Prednisolone was tapered off over a year and was replaced by methotrexate 4 mg/week and mycophenolate mofetil 1000 mg/day. There is no recurrence after two years of follow-up.

Case 2

**Figure 4:**
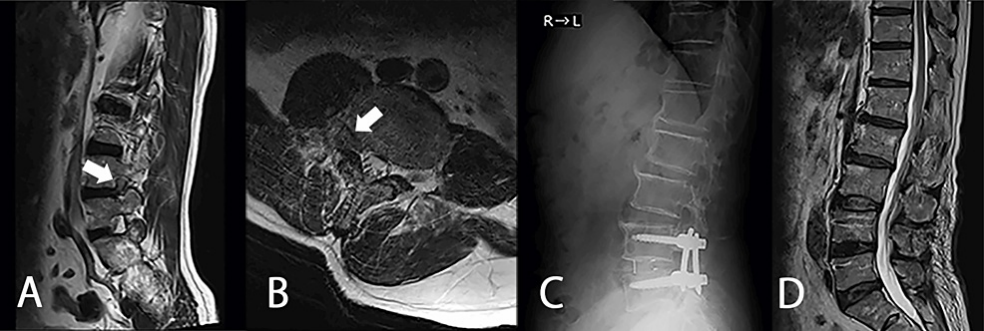
Initial images of case 2 A: T2WI sagittal section; B: T2WI axial section. Initial MRI scans of case 2 showed a herniated disc in RT. L4/5 intervertebral foramen (white arrows). C: Lateral lumbar X-ray, D: T2WI sagittal section. Images after the TLIF operation MRI: magnetic resonance imaging; TLIF: transforaminal lumbar interbody fusion

**Figure 5:**
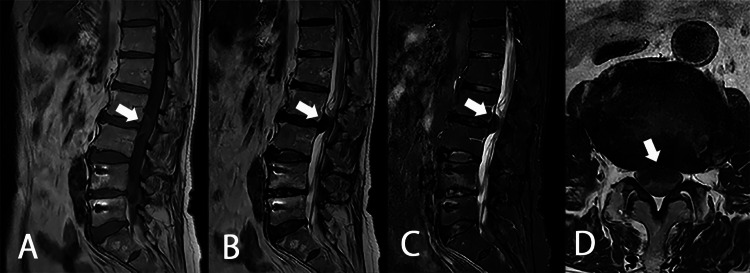
MRI scans of case 2, three years after the first TLIF operation A: T1WI sagittal section; B: T2WI; C: STIR; D: T2WI axial section. Extramedullary mass located in L2/3 intervertebral disc level (white arrows). The initial radiological diagnosis was a herniated disc MRI: magnetic resonance imaging; TLIF: transforaminal lumbar interbody fusion; STIR: short tau inversion recovery

A 67-year-old male with a history of L4/5 lumbar disc herniation (Figure 4A, 4B), for which he underwent transforaminal lumbar interbody fusion (TLIF) surgery three years prior (Figure 4C, 4D), presented with bilateral thigh pain 2.5 years post-operation. At that time, a mildly elevated inflammatory response was observed (WBC 11900/μl, CRP 7.68 mg/dl) (Table 2). MRI revealed a mass resembling a disc herniation at the L2/3 intervertebral level. Six months later, the thigh pain persisted, and the mass increased in size (Figure 5). The differential diagnosis based on MRI was a herniated disc; however, the imaging findings suggested the possibility of a neoplastic lesion.

**Table 2:** Laboratory findings of case 2 OP: operation; IST: immunosuppressive therapy; AST: aspartate aminotransferase; ALT: alanine aminotransferase; BUN: blood urea nitrogen; MPO-ANCA: myeloperoxidase anti-neutrophil cytoplasmic antibodies; PR3-ANCA: proteinase-3-anti-neutrophil cytoplasmic antibodies; sIL2R: soluble IL2 receptor; WBC: white blood cell; RBC: red blood cell; CSF: cerebrospinal fluid

Investigations	Initial checkup	After OP	After IST	Normal range
Biochemistry/immunology				
Total protein (g/dL)	7.1	7.1	6.3	6.6-8.1
Serum albumin (g/dL)	3.8		3.8	4.1-5.1
AST (U/L)	18	21	22	7-38
ALT (U/L)	12	16	27	4-43
BUN (mg/dL)	14.5	19.5	20.4	8.0-20.0
Serum creatinine (mg/dL)	1.13	0.93	1.08	0.60-1.20
C-reactive protein (mg/dL)	7.68	0.65	0.09	0-0.3
IgG (mg/dL)		1317	670	820-1,400
IgA (mg/dL)		470		90-400
IgM (mg/dL)		58		52-270
IgG4 (mg/dL)		70		11-121
MPO-ANCA (U/mL)		0.2		<3.5
PR3-ANCA (U/mL)		<0.6		<3.5
sIL2R (U/mL)		536		122-496
Complete blood cell count				
WBC (/µL)	11,900	8,800	9,200	3,600-8,000
Neutrophil (%)	80	71	68	37.0-72.0
Lymphocyte (%)	13	20	27	19.0-49.0
Monocyte (%)	5	6	5	2.0-11.0
Eosinophil (%)	2	3	0	0-5.0
RBC (×10^4^/µL)	420	431	379	380-480
Hemoglobin (g/dL)	13.8	14.0	12.6	12.0-16.0
Hematocrit (%)	40.8	41.1	37.9	36.0-48.0
Platelet (×10^4^/µL)	19.2	24.2	25.5	12.0 -40.0
CSF cell count and chemistry				
Cell		78	17	<5
RBC		0	0	
Protein		637	138	<40
Glucose		59	68	50-70
IgG (mg/dL)		124.0	6.6	
IgG index		0.82	0.51	0-0.73
High-sensitivity IL-6 (pg/mL)		93.37	2.64	
IgG4 (mg/dL)		58	<5	

Initially, L2-L3 posterior decompression and discectomy were planned, but we could not visualize the mass and encountered difficulties with the extradural approach. Hence, a dural incision was made, and the cauda equina nerves were exposed. We observed a mass lesion between the nerves and found that it was not a herniated disc but was continuous with the dura mater. The adhesions between the lesion and the cauda equina nerves were mild. The lesion was removed as much as possible.

**Figure 6:**
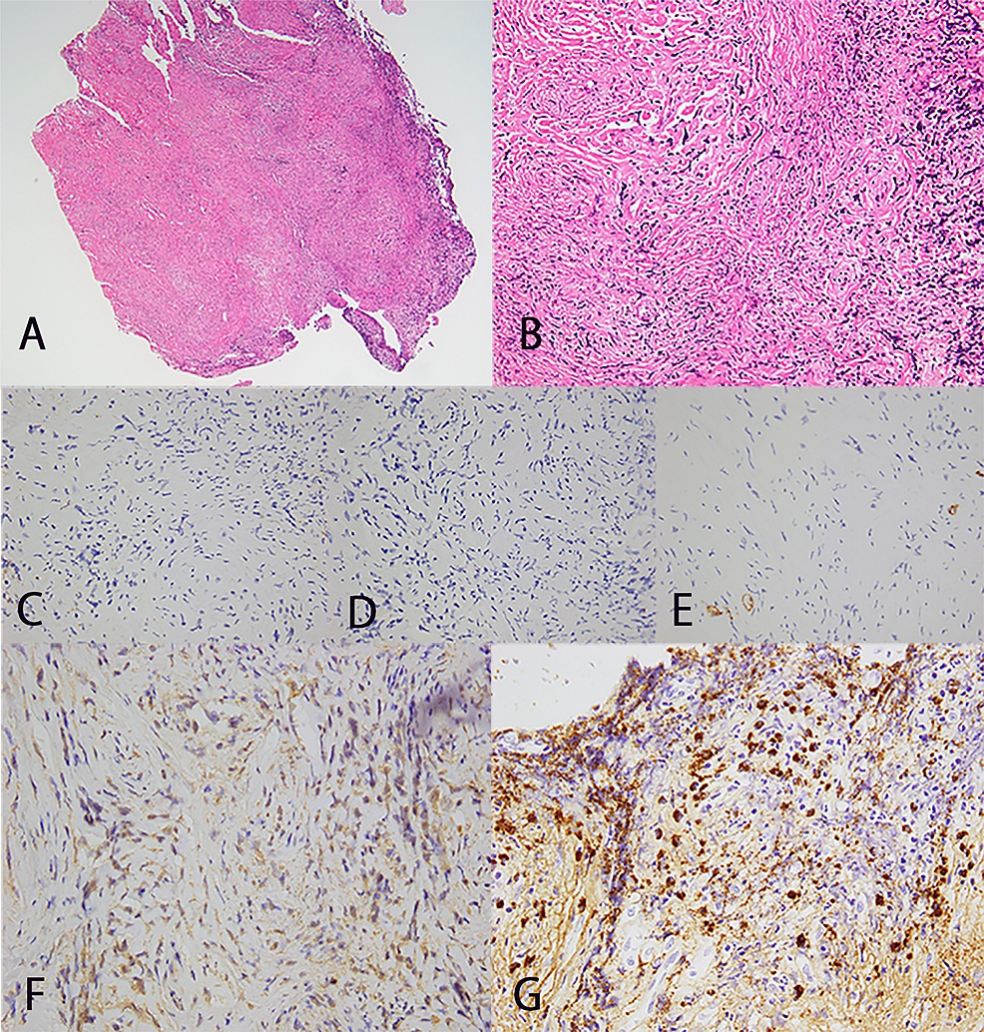
Specimen of mass lesion of case 2 Hematoxylin-eosin stain. A: weak magnification (40×); B: strong magnification (400×). EMA, PgR, and CD34 staining were negative (C: EMA, D: PgR, E: CD34). IgG4-positive cells are observed in multiple locations (F, G)

Pathologically, there was a proliferation of spindle-shaped cells with a background of relatively mature fibrous tissue and scattered lymphocytic and plasma cell infiltrates. The cell density was low, and cellular dysmorphism was mild. No mitosis, necrosis, or phlebitis obliterans was observed (Figure 6A, 6B). Immunostaining was performed to differentiate between fibrous meningioma, solitary fibrous tumor, and IgG4-RD (Figure 6C, 6D, 6E). The patient had multiple positive IgG4-positive cells, and the ratio of IgG4-positive cells/CD138-positive cells was >40% at the same site (Figure 6F, 6G). The pathological findings revealed probable histological features of IgG4-RD. IgG4-RD can cause pancreas, kidneys, and salivary gland lesions. However, no lesions other than those in the spinal canal were noted in this case. Although serologically there was no obvious IgG4 or MPO-ANCA elevation (IgG4 70 mg/dl, MPO-ANCA negative), IgG4/IgG elevation in CSF and pathology led the neurologist to diagnose HP with IgG4-RD (IgG4/IgG in serum: 0.053; IgG4/IgG in CSF: 0.467) (Table 2).

**Figure 7:**
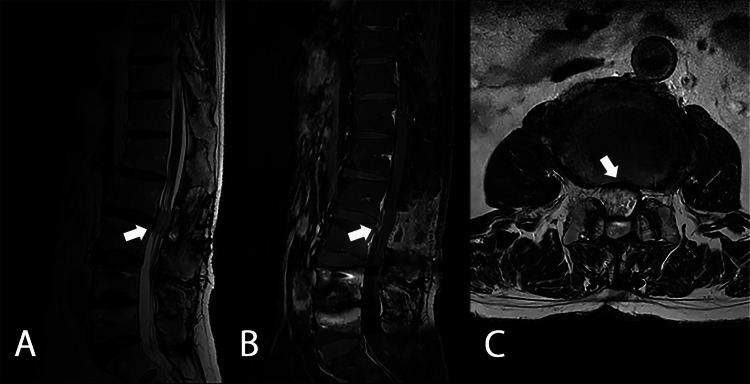
MRI scans three months after the removal of L2/3 mass lesion and immunosuppressive therapy A: T2WI sagittal section; B: T1WI with contrast enhancement sagittal section; C: T2WI axial section. The hypertrophic dural lesion almost disappeared (white arrows) MRI: magnetic resonance imaging

Treatment involved the administration of prednisolone and azathioprine. Because the cauda equina nerves were determined to have been adequately decompressed surgically, the neurologist started treatment with steroids without rituximab as intensive care and added azathioprine as the steroids were tapered off. Methylprednisolone 1000 mg was administered intravenously for three days, followed by oral prednisolone 30 mg/day. Prednisolone was tapered to 5 mg/day over three months, and 100 mg/day of azathioprine was added after two months. The NUDT15 gene codon 139 polymorphism analysis was Arg/Arg, and the occurrence of side effects such as leukopenia and alopecia associated with azathioprine was expected to be rare. Follow-up MRI scans revealed the complete disappearance of the mass three months later (Figure 7). Slight right lower limb paralysis appeared postoperatively but gradually improved, and the patient was discharged with a cane. There is no recurrence after one year of follow-up.

## Discussion

Pachymeningitis is characterized by a partial or diffuse thickening of the dura mater of the brain and spinal cord due to chronic inflammation. Pathology is generally divided into three main categories: idiopathic HP (40.0%), secondary HP with ANCA-associated vasculitis (30.2%), and secondary HP with IgG4-RD (8.8%) [1,3,4]. In a few HP cases, ANCA and IgG4 are combined serologically (3.8%) [1]. Spinal idiopathic HP and HP associated with ANCA-related vasculitis are more common in middle-aged women [6,7]. Spinal HP associated with IgG4-RD was more common in middle-aged men [8].

The initial clinical symptom is frequent back pain. This is often followed by abnormal sensations, muscle weakness, and bowel-bladder dysfunction over several months [6-8]. As in our case, laboratory blood tests may show elevated ESR or CRP levels [5,9]. Both ANCA-associated vasculitis and IgG4-RD often manifest as systemic organ manifestations. ANCA-associated disease is a primary systemic vasculitis with upper respiratory tract, lung, and renal lesions being the most common [9]. IgG4-RD shows the infiltration of IgG4-positive plasma cells and tissue fibrosis. It causes organ enlargement and nodules throughout the body, including the pancreas, thyroid gland, lacrimal gland, salivary gland, and bile ducts [10]. None of our cases had typical ANCA-associated vasculitis nor IgG4-RD lesions other than dural lesions.

According to the diagnostic criteria for IgG4-RD [11], the two cases were diagnosed as possible (case 1) and probable (case 2) IgG4-RD. As in case 2, serum IgG4 levels are normal in 25-30% of patients with confirmed IgG4-RD [10]. IgG4-RD histologically mimics other inflammatory conditions. The presence of storiform fibrosis and an increased number of IgG4-positive plasma cells can be helpful for the diagnosis [12]. IgG4-related HP can be a seronegative central nervous system-localized disease that cannot be diagnosed using only blood samples because of the lack of typical findings of IgG4-RD in systemic organs. IgG4-RD itself was established in 2001. It is possible that some HPs that were previously considered idiopathic were IgG4-RD [13].

In every pathological type, the thoracic level was the most common site of occurrence (58-90%), followed by the lumbar and cervical spine [6-8]. On MRI, HP lesions appear as dura-based masses with low signal intensities on T1- and T2-weighted images, extending over multiple vertebral levels. Contrast-enhanced MRI may show a more intense lesion enhancement [5,14]. The patients included in our study exhibited similar characteristics. Most lesions were circumferential thickenings of the spinal dura mater (37.8%) [15]. However, there are also cases in which the thickened area is more dorsal (33.3%) or ventral (13.7%) to the dural canal, which must be differentiated from other extramedullary lesions of the spinal canal [7,15]. Spinal HP has no clinical or radiological characteristics and can mimic common spinal tumors [12,16]. However, to the best of our knowledge, no report has differentiated it from disc herniation. In the past, there have been some reports of herniated discs that required differentiation from benign tumors [17,18], and it is possible that the initial imaging diagnosis of a tumor-like HP that develops at the level of the disc, as in case 2, could be a herniated disc.

In cases of spinal HP that are already symptomatic, surgery may be performed for histopathological diagnosis and decompression of the spinal cord. In previous reports, surgery was almost always the treatment of choice, especially when idiopathic HP was suspected because of negative markers in the blood or CSF [7]. Surgical treatment is also performed in approximately 70% of autoimmunity-mediated HP cases, such as ANCA-related HP or IgG4-RD [6,8]. Surgical treatment options include durotomy and expansive duraplasty to remove the compression caused by the thickened dura mater and spinal decompression with laminectomy. Immunosuppressive therapy is often administered in cases of autoimmunity-mediated HP. Although there are no standard guidelines for the treatment of IgG4-related HP [13,19], glucocorticoid therapy is usually recommended after confirmation of the diagnosis [13,19]. As in case 1, an open-label study showed an excellent rate of clinical response to rituximab [20]. The concomitant use of immunosuppressive drugs, such as methotrexate, azathioprine, mycophenolate mofetil, or cyclophosphamide, may be an option [13].

## Conclusions

We encountered two cases in which the initial imaging diagnosis was meningioma or herniated disc and the final diagnosis was IgG4-related spinal HP. Spinal HP may mimic spinal tumors or herniated discs on imaging, and the initial symptom is mainly nonspecific back pain, which may lead to misdiagnosis because of the rarity of the disease.

IgG4-related HP, which often responds to immunosuppressive therapy, can be a serologically negative central nervous system-localized type with poor findings in other systemic organs. It can be misdiagnosed as a tumor, degenerative disease, or idiopathic HP without a pathological examination of the hypertrophic dura mater or an examination of IgG4 in the CSF.
